# Impact of sex and gender on COVID-19 outcomes in Europe

**DOI:** 10.1186/s13293-020-00304-9

**Published:** 2020-05-25

**Authors:** Catherine Gebhard, Vera Regitz-Zagrosek, Hannelore K. Neuhauser, Rosemary Morgan, Sabra L. Klein

**Affiliations:** 1 Department of Nuclear Medicine, University Hospital Zurich, Raemistrasse 100, 8091 Zurich, Switzerland; 2 Center for Molecular Cardiology, University of Zurich, Schlieren, Switzerland; 3 Department of Internal Medicine II, Medical University of Vienna, Vienna, Austria; 4 University of Zurich, Zurich, Switzerland; 5 Charité, Universitätsmedizin Berlin, Berlin, Germany; 6 DZHK (German Centre for Cardiovascular Research), partner site Berlin, Berlin, Germany; 7 Robert Koch Institute, Berlin, Germany; 8 Department of International Health, The Johns Hopkins Bloomberg School of Public Health, Baltimore, Maryland USA; 9 W. Harry Feinstone Department of Molecular Microbiology and Immunology, The Johns Hopkins Bloomberg School of Public Health, Baltimore, Maryland, USA

**Keywords:** Gender, Sex, COVID-19, Renin angiotensin aldosterone system, Immune system

## Abstract

**Background:**

Emerging evidence from China suggests that coronavirus disease 2019 (COVID-19) is deadlier for infected men than women with a 2.8% fatality rate being reported in Chinese men versus 1.7% in women. Further, sex-disaggregated data for COVID-19 in several European countries show a similar number of cases between the sexes, but more severe outcomes in aged men. Case fatality is highest in men with pre-existing cardiovascular conditions. The mechanisms accounting for the reduced case fatality rate in women are currently unclear but may offer potential to develop novel risk stratification tools and therapeutic options for women and men.

**Content:**

The present review summarizes latest clinical and epidemiological evidence for gender and sex differences in COVID-19 from Europe and China. We discuss potential sex-specific mechanisms modulating the course of disease, such as hormone-regulated expression of genes encoding for the severe acute respiratory syndrome coronavirus 2 (SARS-CoV2) entry receptors angiotensin converting enzyme (ACE) 2 receptor and TMPRSS2 as well as sex hormone-driven innate and adaptive immune responses and immunoaging. Finally, we elucidate the impact of gender-specific lifestyle, health behavior, psychological stress, and socioeconomic conditions on COVID-19 and discuss sex specific aspects of antiviral therapies.

**Conclusion:**

The sex and gender disparities observed in COVID-19 vulnerability emphasize the need to better understand the impact of sex and gender on incidence and case fatality of the disease and to tailor treatment according to sex and gender. The ongoing and planned prophylactic and therapeutic treatment studies must include prospective sex- and gender-sensitive analyses.

## Introduction

In December 2019, a novel β-coronavirus, now designated SARS-CoV2 (severe acute respiratory syndrome coronavirus 2), was identified as the cause of an outbreak of acute respiratory illness in Wuhan City, China [1]. SARS-CoV2 causes severe respiratory disease, termed coronavirus disease 2019 (COVID-19), which represents its most frequent lethal complication. Since its outbreak, SARS-CoV2 has spread to 196 countries and has been declared a pandemic by the World Health Organization (WHO) on March 11, 2020 [2, 3]. It has caused over 2 million confirmed infections with over 130,000 deaths worldwide (as of April 15, 2020), of which two-thirds have occurred in Europe [4]. To date, no specific antiviral treatment for SARS-CoV2 exists, but a number of investigational agents are currently being explored including remdesivir, lopinavir-ritonavir, a combined protease inhibitor, chloroquine/hydroxychloroquine, colchicine, and tocilizumab, an IL-6 inhibitor [5]. The worldwide case fatality rate of 3.4% of COVID-19 now exceeds that from seasonal influenza [2]. Death results from acute respiratory distress syndrome (ARDS), acute respiratory failure, coagulopathy, septic shock, and metabolic acidosis [6]. Cardiovascular complications of COVID-19 comprise arrhythmias, acute cardiac injury, and shock, and have been reported in 7–17% of hospitalized patients [7]. In Italy, the estimated case fatality rate was 7.2% [8], while it was 0.9% in South Korea [3] and 2.3% in China [6]. Case fatality is highest in those aged > 80 years (14.8% in China, 20.2% in Italy) and in patients with pre-existing conditions including cardiovascular disease, diabetes mellitus, chronic respiratory disease, hypertension, and cancer [6, 9]. Among all comorbidities, cardiovascular disease in the elderly was most consistently associated with adverse outcomes, as a case fatality rate of 10.5% has been reported in this high-risk population [6].

### Sex differences in COVID-19 epidemiology and case fatality

**Fig. 1:**
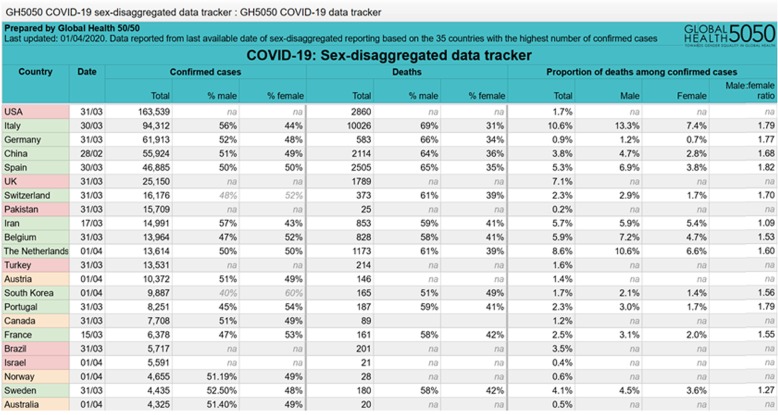
Sex-disaggregated data of confirmed COVID-19 cases and deaths provided by Global Health 50%50 data tracker as of April 2, 2020 [16]

**Fig. 2:**
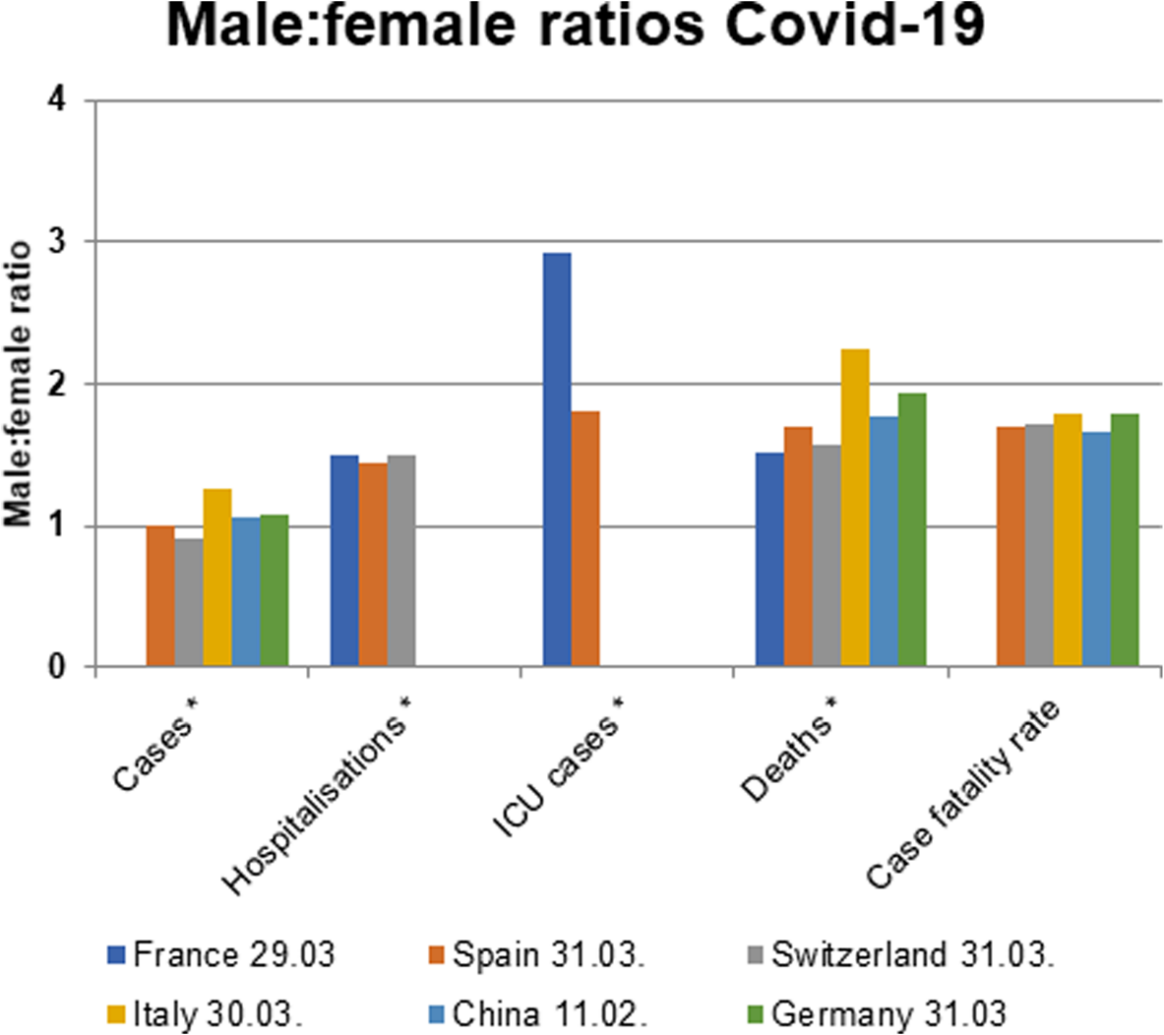
Male to female ratios of COVID-19 cases, hospitalizations, intensive care unit (ICU) admissions, deaths, and case-fatality rates in European countries and China as of April 2, 2020. *absolute numbers are provided. Sex-disaggregated data were not available for all indicators

First reports from China have pointed to a sex imbalance with regard to detected cases and case fatality rate of COVID-19 [1, 10, 11]. However, to date only few reports have addressed the sex disproportion in COVID-19 incidence and disease course and a thorough analysis of underlying causes is currently lacking [12–15]. As the disease has spread across multiple continents, the *Global Health 50/50 research initiative* presented an impressive overview of sex-disaggregated data from countries worldwide clearly demonstrating similar numbers of cases in women and men, but an increased case fatality in men [16] (Fig. 1). Nevertheless, sex-disaggregated data are still not provided by all countries, the interaction of sex and age is usually not visible in the public databases, and number of cases and case fatality vary significantly by region. To obtain a detailed European view and to cover these aspects, we collected latest epidemiological data (as of April 1st) on confirmed COVID-19 cases in Italy, China, Spain, France, Germany, and Switzerland [17–22] across multiple disease metrics including recently published hospitalization and intensive care (ICU) admission data. Similar to global statistics, these reports show no major sex differences in the absolute number of confirmed COVID-19 cases in those countries where sex-disaggregated data were available (Fig. 2). However, equal absolute numbers of cases in women and men may point towards a higher incidence in men in the older age groups (i.e., *proportions* of COVID-19 diagnosed older men among men in that age group) since older men are fewer in absolute numbers than older women due to their shorter life expectancy. In fact, reports from Switzerland and Germany have recently reported incidence rates (cases per 100,000 inhabitants by age and sex), which confirm an increased disease incidence in men > 60 years, [21, 22]. In detail, the disease incidence in men per 100,000 Swiss inhabitants in the age groups of 60–69 years, 70–79 years, and 80+ years was 267, 281, and 477, respectively, as of March 30. The numbers reported in men exceeded the ones reported in women by 74, 87, and 108 per 100,000 Swiss inhabitants, respectively. In Germany, relative differences between men and women were similar to Switzerland, but at a lower level, with the incidence in Germany being one-third of that in Switzerland. It is notable, however, that the number of confirmed cases and therefore also the incidence depends largely on testing strategy in countries and regions.

Novel data on disease course and severity show 50% more hospitalized men than women (Fig. 2). Notably, although the overall number of confirmed COVID-19 cases across all age groups is currently sex balanced in Switzerland, the hospitalizations in men exceed the one observed in women by 1.5-fold. A similar gender distribution in hospitalization rates is observed in France. This imbalance supports a higher susceptibility of men to develop severe respiratory disease following SARS-CoV2 infection, leading to more hospital admissions. While the number of ICU admissions of men and women are currently unknown in Switzerland, in France, and in the Lombardy region (Italy), the number of men receiving ICU care is 3-fold and 4-fold higher than the number of women [23]. The latter might be indicative of gender differences in COVID-19 disease severity; however, gender inequity in ICU admission policies may also play a role.

**Fig. 3:**
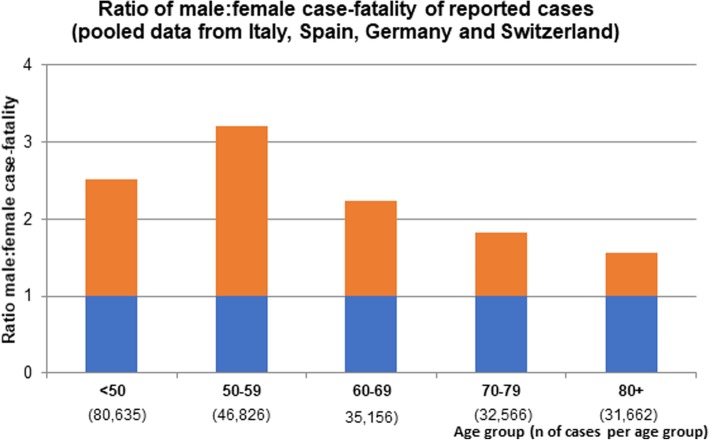
Male predominance in COVID-19 case fatality (deaths divided by confirmed cases) in Italy, Spain, Germany, and Switzerland by age. A male to female mortality ratio of 1 would reflect gender balance, the red bars reflect male predominance. Pooled data from Italy as of March 30, 2020, Spain as of March 31, 2020, Germany as of April 1, 2020, and Switzerland as of March 31, 2020

Significant differences in the male to female COVID-19 case fatality ratio can be observed between European countries. The latter may also reflect the age-sex mix of cases by country as well as national testing strategies, besides case fatality. Nevertheless, case fatality rates reported in China, Italy, Spain, France, Germany, and Switzerland are relatively homogenous and range between 1.7–1.8. This supports the view that a consistent biological phenomenon is operating, accounting for the higher case fatality in men, independent of country-specific demographics and testing strategies (Fig. 2) [17–19, 21, 22]. In addition, pooled data comprising 227,219 confirmed cases and 14,364 deaths suggest that the male to female case fatality ratio is consistently elevated through all age groups and may even be most pronounced at middle age (Fig. 3). The latter is a novel observation which further supports the notion that age as well as gender-specific behavior and/or biological variables interact in COVID-19 disease vulnerability. However, more data are needed to confirm an interaction between age and sex in COVID-19 case fatality.

### Sex differences in ACE2 and TMPRSS2 regulation

To enter cells, SARS-Cov-2 binds to the angiotensin converting enzyme (ACE) 2 receptor and the cellular serine protease TMPRSS2 for priming [24] (Fig. 5). ACE2 is a membrane-bound protein and is expressed in multiple tissues including the cardiovascular system, adipose tissue, gut and kidneys, the central nervous system, and in the lungs [25]. The cell-associated form of ACE2 is required for SARS-CoV virus entry into target cells [26]. ACE2 is cleared from the cells by the metalloproteases ADAM10 and ADAM17 [26, 27]. Some reports indicate that circulating levels of ACE2 are higher in healthy and diabetic men as well as in men with renal disease as compared to women [28]. Others found no sex difference but reported higher ACE2 serum activity in older compared to younger women [29]. In patients with type 1 diabetes, circulating ACE2 activity increases with increasing vascular tone and in the presence of microvascular or macrovascular atherosclerotic disease [30]. Soluble ACE2 is enzymatically active and has modest inhibitory effects on viral infection efficiency [31]. However, these data are not yet coherent and the link between circulating ACE2 and COVID-19 is not clear.

ACE2 plays a crucial role in the renin angiotensin aldosterone system (RAAS) as it opposes the vasoconstrictor actions of angiotensin II by converting angiotensin II to vasodilatory angiotensin 1–7 in different organs. ACE2 regulates the cellular biology of cardiomyocytes, cardiac fibroblasts, and coronary endothelial cells in both heart failure with reduced ejection fraction (HFrEF) and heart failure with preserved ejection fraction (HFpEF) models and after experimental myocardial infarction [32, 33]. Therefore, increasing ACE2 activity was considered a potential therapeutic option for COVID-19 [34]. However, a previous report suggests that high protein expression of ACE2 receptor in specific organs was associated with organ failure in patients infected by SARS in 2002/2003 [35], while 35% of myocardial tissue samples of patients who died from SARS showed a reduced myocardial ACE2 protein expression along with viral RNA [36]. A loss of ACE2 function through endocytosis and activation of proteolytic cleavage following SARS-CoV-2 binding has recently been described and could reconcile these apparently contradictory findings [25].

In the lung, ACE2 is primarily expressed in bronchial transient secretory cells or type II alveolar cells [37]. Experimental evidence derived from murine and rat models suggests a protective role of ACE2 activators in vascular remodeling during pulmonary hypertension, in allergic airway inflammation associated with asthma, and in the reduction of pulmonary fibrosis [38, 39]. Further, ACE2 activation improved pulmonary endothelial function in a rat model of pulmonary hypertension via the endothelial nitric oxide synthase (eNOS) pathway and seems to play an important role in smoking-induced lung injury [40]. Indeed, the latter was associated with a significant reduction of ACE2 expression in lung tissue which was reversed by Losartan treatment [41]. These preclinical studies suggest a protective role and a potential therapeutic use of ACE2 in a variety of pulmonary diseases. It is however currently unclear whether the role of ACE2 in pulmonary pathologies differs by sex. In addition to the above mentioned studies, ACE inhibitors and angiotensin receptor blockers (ARBs) have been reported to upregulate ACE2 expression in different organs in humans [42, 43] and experimental animals [44], whereas no effect of ACE inhibitors or ARBs on ACE2 activity was found in other reports [33]. The interaction between COVID-19 and ACE inhibitors or ARBs in patients with heart disease was recently reviewed [45]. This topic is out of the scope of the present review which focuses on sex differences.

**Fig. 4:**
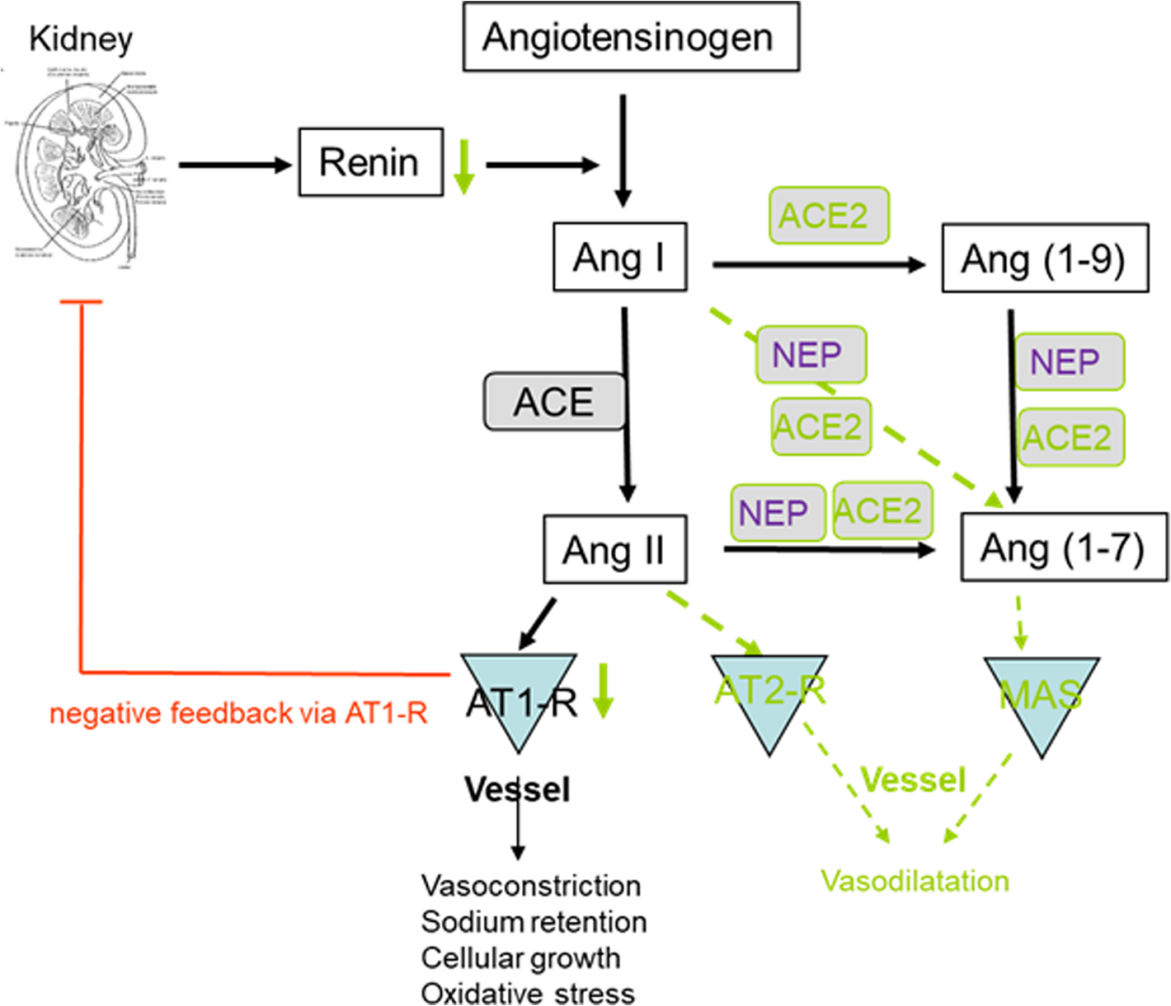
Estrogen and sex regulate components of the renin angiotensin aldosterone system (RAAS). Estrogen-regulated pathways are depicted in green. AT2R angiotensin II type 2 receptor, ACE2 angiotensin converting enzyme 2, NEP neutral endopeptidase neprilysin

There is increasing evidence that sex and sex hormones affect many components of the circulating as well as tissue-based RAAS including ACE2 [46–50] (Fig. 4). Downregulation of angiotensin II receptor type 1 (AT1R) by estrogens, and regulation of renin activity by estrogens have been described and reviewed elsewhere [51, 52]. More recently, it was shown that estrogen modulates the local RAAS in human atrial myocardium via downregulation of ACE and simultaneous upregulation of ACE2, AT2R, and MAS expression levels [53]. The ACE2/Ang1-7/Mas receptor axis appears to be of greater relevance in women than in men [47]. Indeed, genes coding for ACE2 and angiotensin II receptor 2 (AT2R) are located on the X chromosome suggesting a potential for higher expression in women [54]. Nevertheless, reports from a number of preclinical studies agree that ACE2 is frequently higher expressed in males than in females, mainly under pathological conditions [47, 50, 55].

In addition to sex chromosome complement, sex hormones promote opposite effects on ACE and ACE2 activity, cardiac hypertrophy, and contractility in spontaneously hypertensive rats [56]. Ovariectomy led to increased ACE2 activity in females, whereas in males, orchiectomy decreased ACE2 activity. In agreement with these data, ovariectomy increased ACE2 expression in the female kidney, and adipose tissue, and estradiol replacement reduced ACE2 expression [46]. Thus, testosterone seems to maintain high ACE2 levels in the heart and kidney, whereas estrogen reduces ACE2 expression in these organs. Based on these data, we must assume that a significant interaction between sex hormones and ACE2 expression exists.

In humans, several clinical trials highlight the relevance of sex differences in the RAAS. In fact, a recent prospective cohort study indicates that women require lower doses of ACE inhibitors for heart failure treatment than men [57]. Also, the neprilysin (NEP) inhibitor sacubitril, which degrades angiotensin peptides, in combination with valsartan, has recently been shown to exert beneficial effects in women with HFpEF, but less so in men [58]. Unfortunately, specific mechanisms accounting for this difference have not been reported in these studies. A higher tissue expression of ACE2 has been observed in Asian men as compared to women [28, 59], while in our own unpublished investigation in tissue samples from patients with aortic valve stenosis, ACE2 was upregulated 4–5 fold in the myocardium of men as compared to their female counterparts. In contrast, no sex difference in ACE2 expression was seen in control hearts [60]. Whether these sex differences in ACE2 regulation are of clinical relevance remains to be determined.

**Fig. 5:**
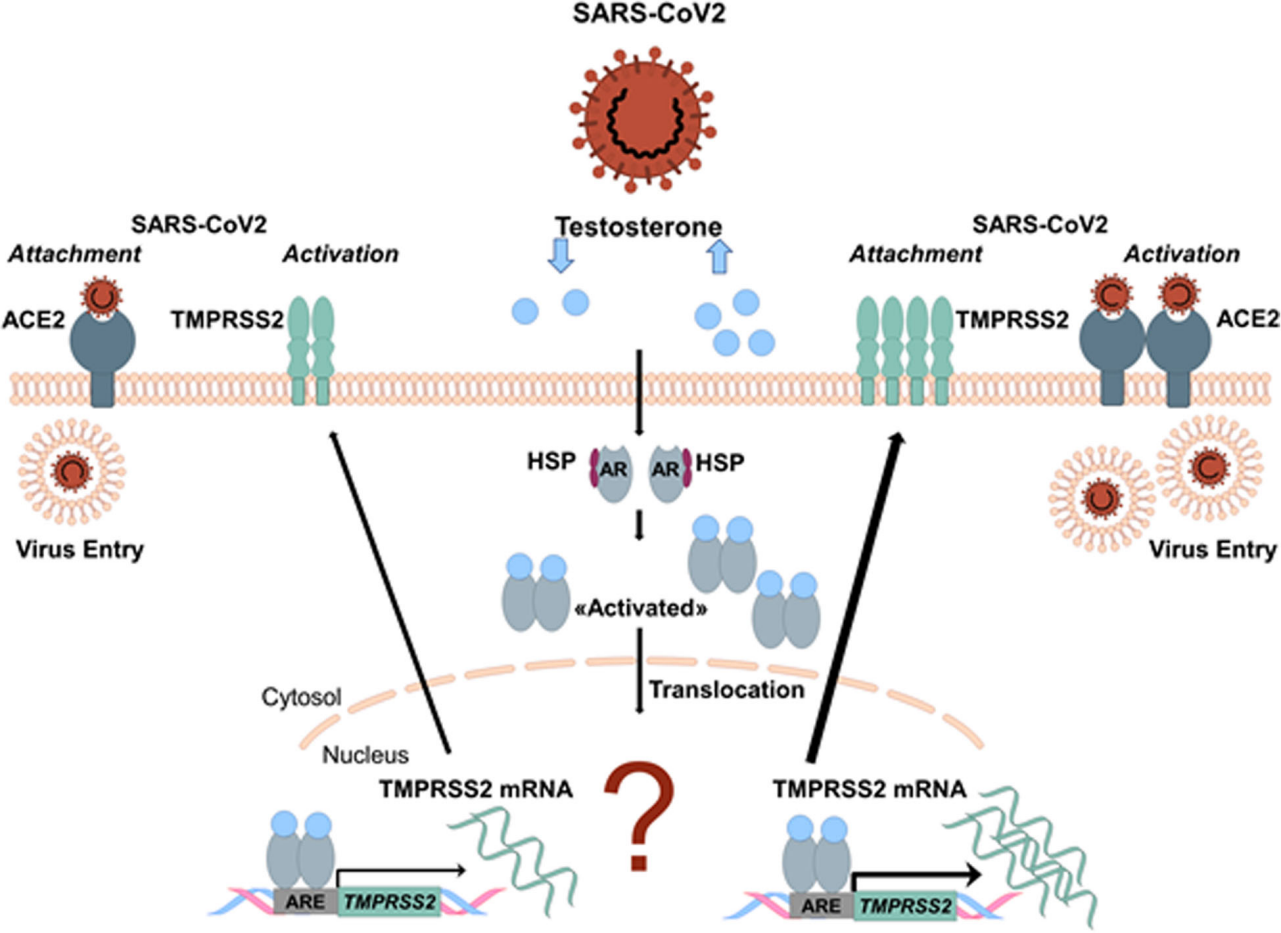
Sexual dimorphism in TMPRSS2-mediated SARS-CoV2 host cell entry. Androgen receptors (ARs) are activated via heat shock proteins (HSPs) release in response to changes in intracellular testosterone concentration. ARs are then phosphorylated and translocated as homodimers into the nucleus, prompting transcriptional activation of TMPRSS2 and translation of the TMPRSS2 protein [149]. At the cell membrane, TMPRSS2 facilitates viral entry and spreads into the host cell by activating the spike proteins [24]

The second protein, necessary for SARS-CoV2 invasion into cells, the cell-surface serine protease TMPRSS2 is predominantly expressed in prostate epithelium, in high-grade prostate cancers, and in the majority of human prostate cancer metastases [61, 62]. Although TMPRSS2 is expressed several fold higher in the prostate relative to any other human tissue, the serine protease has also been detected in airway epithelia where its normal physiologic function remains unknown [63]. TMPRSS2 transcription is regulated by androgenic ligands and an androgen receptor binding element in the promoter [64] (Fig. 5). Notably, recurrent gene fusions of the 5′ untranslated region of TMPRSS2 to the transcription factor ERG is the most frequent genomic alteration in early- and late-stage prostate cancer and results in overexpression of ERG. The latter is present in both early- and late-stage prostate cancer [64]. However, it is currently unclear under which conditions the fusion protein is generated, whether TMPRSS2 is also regulated by estrogen, and whether it plays a role in COVID-19. The involvement of TMPRSS2 in viral S protein priming might explain, at least in part, the higher case fatality seen in males affected by COVID-19. Accordingly, a TMPRSS2 inhibitor has recently been shown to block entry of the virus in vitro and might become a therapeutic strategy for antiviral intervention [24]. Whether previous prostate cancer and anti-androgenic treatment might affect virus entry and the course of disease is currently unknown [64].

### Sex differences in immune responses to viruses

Females and males differ in their susceptibility and response to viral infections, leading to sex differences in incidence and disease severity [65]. For infectious diseases caused by viruses, there are numerous and diverse ways in which sex and gender can impact differential susceptibility between males and females. For example, human studies reveal that females have over 40% less human immunodeficiency virus (HIV) RNA in circulation than males. Despite having less circulating HIV RNA than males, females who are matched with males on their HIV RNA loads have a 1.6-fold higher risk of developing AIDS [66]. Although exposure to influenza A viruses is often higher in males, fatality following exposure to pathogenic influenza A viruses is reportedly higher in females [67]. In contrast, the prevalence of serum hepatitis B virus (HBV) surface antigen, HBV DNA titers, and development of hepatocellular carcinoma is higher in males than females [68–70].

The innate recognition and response to viruses as well as downstream adaptive immune responses during viral infections differ between females and males. The number and activity of innate immune cells, including monocytes, macrophages, and dendritic cells (DCs) as well as inflammatory immune responses in general are higher in females than in males [71–73]. Toll-like receptor (TLR) 7 is a pattern recognition receptor in the endosomes of several immune cells, including plasmacytoid DCs and B cells, and is used to detect single stranded RNA viruses, including coronaviruses. The TLR7 gene, encoded on the X chromosome, may escape X inactivation resulting in higher expression levels of TLR7 in females when compared to males [74–76]. Exposure of peripheral blood mononuclear cells (PBMCs) to TLR7 ligands in vitro causes higher production of interferon-α (IFNα) in cells from females than from males [77], and plasmacytoid DCs (pDCs) from females and female mice have higher basal levels of IFN regulatory factor 5 (IRF5) and IFNα production following TLR7 ligand stimulation [78]. Immune responses to viruses can vary with changes in sex hormone concentrations naturally observed over the menstrual cycle, following contraception, after menopause and during hormone replacement therapy (HRT) as well as during pregnancy [79].

With regard to adaptive immune responses, females generally exhibit greater humoral and cell-mediated immune responses to antigenic stimulation, vaccination, and infection than do males [80]. Both basal levels of immunoglobulin [81] as well as antibody responses are consistently higher in females than in males [82]. In humans, global analysis of B cell gene expression signatures reveals that the majority of genes differentially expressed between the sexes are significantly upregulated in B cells from adult females compared with males [83]. Clinical studies reveal that males have lower CD3+ and CD4+ cell counts, CD4+:CD8+ cell ratios, and helper T cell type 1 (Th1) responses than females [84–87]. Females also exhibit higher cytotoxic T cell activity along with upregulated expression of antiviral and pro-inflammatory genes, many of which have estrogen response elements in their promoters [88].

Sex steroids, particularly testosterone (T), estradiol (E2), and progesterone (P4), influence the functioning of immune cells. Sex steroids alter the functioning of immune cells by binding to specific receptors, which are expressed in various lymphoid tissue cells as well as in circulating lymphocytes, macrophages, and dendritic cells [89]. The binding of sex steroids to their respective steroid receptors directly influences cell signaling pathways, including NF-κB, cJun, and interferon regulatory factor (IRF) 1, resulting in differential production of cytokines and chemokines [89]. Although direct effects of gonadal steroids cause many sex differences in immune function, some sex differences might be caused by the inherent imbalance in the expression of genes encoded on the X and Y chromosomes [90]. Polymorphisms or variability in sex chromosomal genes as well as in autosomal genes that encode for immunological proteins can also contribute to sex differences in immune responses [91].

Sex differences in immune response in cardiac tissues also depend on age. We have recently shown that females develop stronger chronic immune reactions in the myocardium with old age [92]. Aging is associated with the development of a chronic low-grade inflammatory phenotype (CLIP) [93]. Such CLIP may be induced by chronic viral infections, among others. Cellular senescence may also contribute to CLIP as senescent cells circulate in the tissues through the body. They secrete a variety of pro-inflammatory mediators, stimulating CLIP. Furthermore, factors as smoking, decreased production of sex steroids, and accumulation of adipose tissue may also contribute to CLIP.

### Gender-related risk factors and impact

When considering differentials in incidence and case fatality between males and females, we must also consider how sex intersects with gender to influence vulnerability. Gender is defined as the social and cultural norms, roles, attributes, and behaviors that a society considers appropriate for men and women or boys and girls [94]. Evidence suggests that the current COVID-19 pandemic has both primary and secondary effects related to sex and gender. Primary effects include differences between males/men and females/women in incidence and case fatality, while secondary effects include differences in social and economic consequences as a result of the pandemic, including risk of domestic violence [95, 96], economic and job insecurity, and increased domestic workload [15].

Preliminary data indicate an association between comorbidities, such as chronic lung disease, hypertension, and cardiovascular disease, and severity of COVID-19 [16]. Worldwide, these morbidities are higher among men than women [97], except for older age groups. Gender differences in risk behaviors, such as smoking and drinking, may be contributing to the gender gaps in mortality of such non-communicable diseases [13]. Smoking and drinking rates are higher among men than women worldwide. Such behaviors are associated with the risk of developing comorbidities [16]. These behaviors are linked to gender norms related to what is considered appropriate behaviors and activities for men and women [98]. Other gendered norms and behaviors which may be contributing to a higher incidence among men include lower rates of hand washing, which is a recognized preventative measure, and delayed healthcare seeking [16]. Evidence from China suggests that patients whose diagnoses were delayed were at greater risk of dying [99]. In this regard, greater system delays between onset of symptoms and initiation of treatment have been described in women with cardiovascular disease [100]; however, no data on prehospital delays in COVID-19 are currently available. Thus, it is currently unknown whether potential gender differences in prehospital delays impose disadvantages on women. Other gendered differences which place women and men at differential risk of infection and/or mortality include rejection of social isolation, social obligations, psychological stress, low quality of life, and low socioeconomic status among COVID-19 [13]. A careful analysis of a patient’s history including traditional cardiovascular risk factors, socioeconomic status, menopausal status, age at menopause, number of pregnancies, pregnancy-related complications, fertility treatments, postmenopausal HRT, hormonal contraception, history of breast or prostate cancer, and aromatase inhibitors/anti-androgenic treatments will be essential to discover mechanisms accounting for the gender disparities in COVID-19.

Women’s roles as caregivers—both within the health system and at home—may place them at increased risk of infection. Approximately 70% of health and social care workforce worldwide are women [101], including frontline healthcare workers. Women are also more likely to care for children or other relatives who are ill [15]. Overall, more research is needed to understand how sex and gender, and the intersection of sex and gender, is causing differential outcomes and effects related to COVID-19 among and between men and women. In particular, there is a need to evaluate the influence of such gender variables on disease manifestation and outcomes.

### Sex differences in COVID-19 treatment approaches

Vaccines are the best prophylactic treatment for infectious diseases as they provide immunity and protection prior to infection. Sex and gender impact vaccine acceptance, responses, and outcomes. Females are often less likely to accept vaccines, but once vaccinated, develop higher antibody responses (i.e., primary correlate of protection) and report more adverse reactions to vaccines than males (Table 1) [80]. For example, after vaccination against influenza, yellow fever, rubella, measles, mumps, hepatitis A and B, herpes simplex 2, rabies, smallpox, and dengue viruses, protective antibody responses are twice as high in adult females as compared with males [80]. Data from inactivated influenza vaccines indicate that adult (18–45 years of age) females develop greater IL-6 and antibody responses than males, with diminished differences between the sexes among aged individuals (65+ years of age) [128]. Reduced male-female differences in immune responses to the monovalent 2009 H1N1 vaccine among aged individuals is partly due to reproductive senescence in females, in which higher circulating estradiol concentrations in females are associated with greater antibody responses to the vaccine [128].

**Table 1:** Sex differences in adverse reactions, immune responses, and efficacy of vaccines and antiviral drugs in humans

Virus	Antiviral drug/vaccine	Sex-specific features	Comments	References
HIV	HAART	M < F	CD4+ T cell count, adverse reactions, fat accumulation, drug concentration, virus clearance, hepatitis	[102–108]
	HAART	M > F	Fat loss, survival	[103, 109]
HSV-2	HSV-2 gD vaccine	M < F	Humoral immune responses, cell-mediated immune responses, vaccine efficacy	[110–112]
	Acyclovir	M < F	Frequency of prescription, adverse reaction	[113, 114]
	Acyclovir	M > F	Reduction of virus shedding	[114]
HBV	HBV vaccine	M < F	Humoral immune responses	[115–118]
HCV	Pegylated interferon alpha/ribavirin	M < F	Adverse reaction, sustained virologic response^1^	[119–121]
Seasonal influenza viruses	TIV vaccine	M < F	Humoral immune responses, adverse reactions	[122–125]
	Oseltamivir	M < F	Drug clearance and metabolism^2^	[126]
	Oseltamivir	M > F	Alleviation of symptoms, reduction of viral load	[127]
	Zanamivir	M = F	Alleviation of symptoms, reduction of viral load	[127]

*HAART* highly active antiretroviral therapy, *HBV* hepatitis B virus, *HCV* hepatitis C virus, *HIV* human immunodeficiency virus, *HSV* herpes simplex virus, *TIV* trivalent inactivated influenza virus. ^1^premenopausal females only, ^2^tested in neonates only

For treatment of COVID-19, a number of investigational agents are currently being explored including remdesivir, lopinavir-ritonavir, a combined protease inhibitor, chloroquine/hydroxychloroquine, colchicine, and tocilizumab, an IL-6 inhibitor [5]. Although some of these compounds have shown promise in inhibiting the growth of SARS-CoV2 in vitro [5, 129–131, 132], their “off-label” use carries the risk of adverse side effects such as cardiac arrhythmias and sudden cardiac death [133, 134]. In particular chloroquine and hydroxychloroquine, both antimalarial agents inhibiting the cell entry of SARS-CoV2 by under-glycosylation of ACE2 receptors [129, 130], are known to trigger life-threatening polymorphic ventricular tachycardia (torsades de pointes) by prolonging the heart rate-corrected QT (QTc) interval [134, 135]. Previous reports indicate that women are more prone to develop drug-induced torsades de pointes than men, with 65–75% of drug-induced torsades de pointes occurring in women [136]. Indeed, there are substantial sex differences in the electrocardiographic pattern of ventricular repolarization with a longer QTc interval at baseline being observed in women [48, 136, 137]. Protective effects of testosterone have been suggested to account for the shorter QTc interval and the reduced incidence of drug-induced torsades de pointes in men. However, mechanisms underlying these differences are not fully understood. In addition, experimental and clinical studies have shown that chloroquine exerts different effects on adrenocortical function in female and male rats [138] and depresses testosterone secretion and sperm count in men [139]. The latter is of particular interest in the treatment of COVID-19 as the expression of TMPRSS2, a protein that primes SARS-CoV-2 entry into cells, is upregulated by androgens [140]. The latter has been suggested to account for the higher mortality seen in men affected by COVID-19. However, whether anti-androgenic treatment might affect virus entry and the course of disease is currently unknown.

Further, there is evidence that women encounter more often adverse drug reactions to antiviral treatment than men (Table 1). In addition, pharmacokinetics and treatment responses to antiretroviral therapy with ritonavir and lopinavir differ between males and females [141]. In fact, higher plasma concentrations of ritonavir and a higher total cholesterol:high-density lipoprotein (HDL) ratio have been reported in girls [141, 142], while an atazanavir plus ritonavir regimen was associated with a higher risk of virologic failure in women as compared to men [131].

The current off-label use of anti-inflammatory drugs, such as colchicine, for the reduction of excessive inflammation caused by SARS-CoV2 is also notable. The COLCORONA trial has just started recruiting patients with COVID-19 and will determine whether short-term treatment with colchicine reduces the rate of death and lung complications related to COVID-19 (https://clinicaltrials.gov/ct2/show/NCT04322682). The drug has recently regained popularity when it was shown that colchicine reduced the risk of ischemic cardiovascular events in patients with a recent myocardial infarction [143]. However, while the primary efficacy composite endpoint was reduced by colchicine in the total cohort and in men, a subgroup analysis pointed to a lower efficacy in women [143]. Also, previous experimental work in rats reports a higher acute oral toxicity of colchicine in females as compared to males with female rats being two times more susceptible to the lethal effects of colchicine than male rats [144]. Thus, a sex-specific analysis in the COLCORONA trial will be essential in order to take these differences into account.

Taken together, these data emphasize the importance to consider the effect of age, reproductive status, and exogenous hormonal manipulation when antiviral and other treatment strategies are applied to COVID-19 patients.

## Conclusion

The sex and gender disparities observed in COVID-19 vulnerability emphasize the need to understand the impact of sex and gender on incidence and case fatality of the disease and to tailor treatment according to sex and gender. Experiences from past outbreaks and pandemics have clearly shown the importance of incorporating a sex and gender analysis into preparedness and response efforts of health interventions [67, 145–148]. Policies and public health efforts, however, have not yet addressed the gendered impacts of disease epidemics, outbreaks, or pandemics. Some countries have not disaggregated data by sex and age the way other countries have. In conclusion, governments in all countries should disaggregate and analyze data for sex and age differences. Furthermore, as prophylactic and therapeutic treatment studies begin, inclusion of sex and gender analyses in their protocols must occur.

## Data Availability

Not applicable

## Abbreviations

ARs: Androgen receptors
ARDS: Acute respiratory distress syndrome
ACE: Angiotensin converting enzyme 2
ARBs: Angiotensin receptor blockers
AT2R: Angiotensin II receptor
CLIP: Chronic low-grade inflammatory phenotype
COVID-19: Coronavirus disease 2019
DCs: Dendritic cells
HAART: Highly active antiretroviral therapy
HFpEF: Heart failure with preserved ejection fraction
QTc: Heart rate-corrected QT interval
HSPs: Heat shock proteins
Th1: Helper T cell type 1
HBV: Hepatitis B virus
HCV: Hepatitis C virus
HSV: Herpes simplex virus
HDL: High density lipoprotein
HIV: Human immunodeficiency virus
ICU: Intensive care unit
NEP: Neutral endopeptidase
HRT: Postmenopausal hormone replacement therapy
SARS-CoV2: Severe acute respiratory syndrome coronavirus 2
TIV: Trivalent inactivated influenza virus
TLR: Toll-like receptor
RAAS: Renin angiotensin aldosterone system
WHO: World Health Organization
